# The Role of Carotid-Femoral Pulse Wave Velocity in a Metabolic Syndrome Patient with Sudden Cardiac Arrest: A Case Report

**DOI:** 10.3390/healthcare12040491

**Published:** 2024-02-18

**Authors:** Hau Kim Choy, Paweł Bogdański, Damian Skrypnik

**Affiliations:** 1 Faculty of Medicine, Poznań University of Medical Sciences, 61-701 Poznań, Poland; choyhaukim@gmail.com; 2 Department of Treatment of Obesity, Metabolic Disorders and Clinical Dietetics, Poznań University of Medical Sciences, 60-569 Poznań, Poland

**Keywords:** metabolic syndrome, sudden cardiac death, pulse wave velocity, carotid-femoral pulse wave velocity

## Abstract

Introduction: Carotid-femoral pulse wave velocity reflecting aortic stiffness could be used as an independent predictor of future cardiovascular events for an individual with metabolic syndrome. However, the routine use of carotid-femoral pulse wave velocity is suboptimized in clinical practice. We report a case of metabolic syndrome with increased carotid-femoral pulse wave velocity and subsequently developed myocardial infarction and sudden cardiac arrest. Case presentation: A Polish man of an age between 40 and 50 years previously diagnosed with metabolic syndrome with essential hypertension, obesity, dyslipidaemia, and impaired glucose level. He developed myocardial infarction, ventricular fibrillation, and was successfully resuscitated with defibrillation. The patient showed high–normal traditional cardiovascular risk factors but an increased carotid-femoral pulse wave velocity. The increased carotid-femoral pulse wave velocity is associated with an increased arterial stiffness, which altered the myocardial perfusion and induced the anterior-lateral ST elevation myocardial infarction. The patient actively participated and completed the phase II cardiac rehabilitation programme. To the best of our knowledge, there have been few studies on carotid-femoral pulse wave velocity screening for patients with metabolic syndrome. Pulse wave velocity screening by a physician appears to be helpful in identifying the potential high-risk population with borderline traditional cardiovascular risk factors. Conclusion: This trajectory highlights the clinical relevance of using carotid-femoral pulse wave velocity as an adjunct marker to assess the risk of cardiovascular event for patients with metabolic syndrome.

## 1. Introduction

Pulse wave velocity (PWV) is defined as the propagation speed of the pressure wave through the arterial system which is inversely proportional to arterial compliance. It can be measured using two different non-invasive approaches including carotid-femoral pulse wave velocity (CfPWV) and brachial-ankle pulse wave velocity (BaPWV). In clinical practice, CfPWV is calculated as the distance divided by the time for the pulse wave to travel between the surface point of a suprasternal notch to the femoral [1]. It has emerged as the gold standard method to measure arterial stiffness [2,3]. An elevation of CfPWV is an essential indicator for vessels and target organ damage [4].

Metabolic syndrome (MetS) is a clustering of metabolic abnormality including obesity, dyslipidaemia, hypertension, and insulin resistance. It accounts for a 50–70% increased risk of sudden death regardless of any previous cardiovascular event [5,6]. In Poland, the prevalence of MetS has risen dramatically from 22.6% to 33% in women and 18% to 39% between 2001 and 2014 [7]. The increasing trend of MetS in Poland is mainly due to smoking, obesity, and hypertension. Polish experts proposed that new criteria of MetS should include the core element, obesity, and two of the other elements including elevated blood pressure, abnormal glucose metabolism or elevated non-high-density lipoprotein, and cholesterol [8]. They also suggested that an early detection and intervention could reduce the deterioration of individual components of MetS. Despite PWV being recommended for primary screening for hypertension, it is not a common clinical practice in Poland. Given that MetS increases the risk of myocardial infraction and even sudden cardiac death, an early stringent risk identification is necessary. Our report aims to provide insight on implementing CfPWV screening for asymptomatic patients with MetS to improve cardiovascular risk stratification.

## 2. Patient Information

### 2.1. History

**Figure 1:**
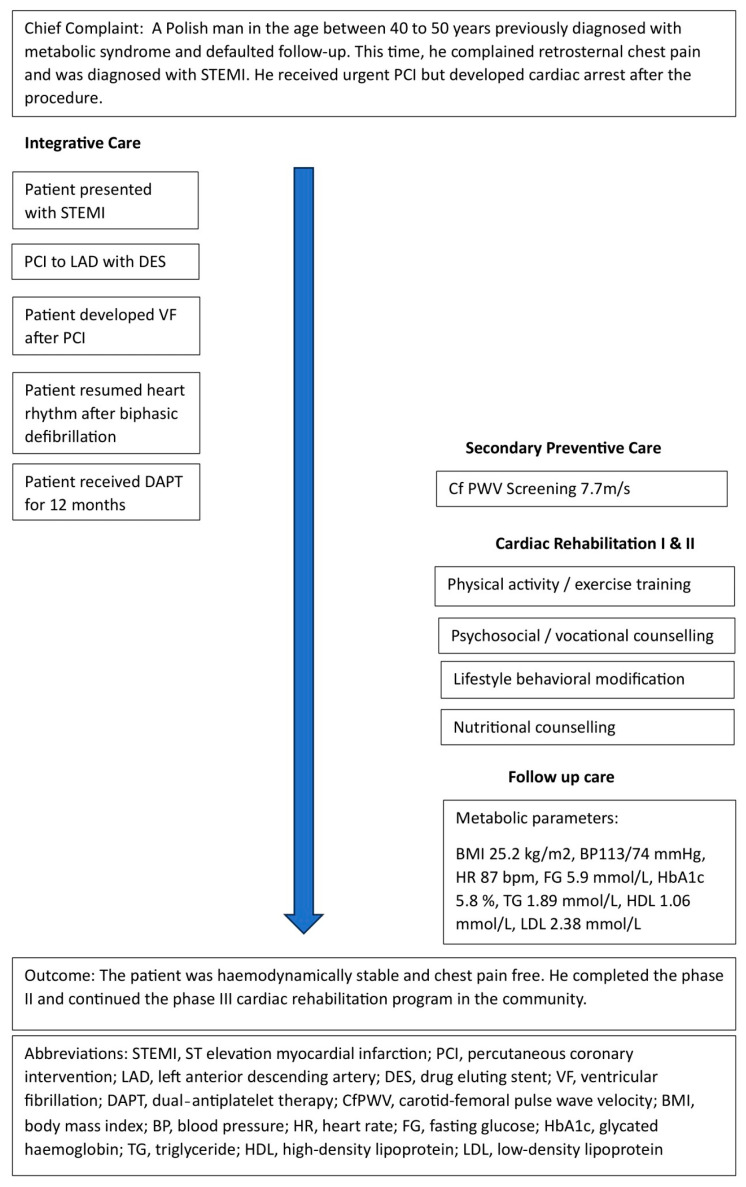
Timeline of patient care.

A Polish male factory worker of an age between 40 and 50 years presenting with retrosternal chest pain was admitted to the emergency department, Poznan, Poland. He had a medical history of MetS with the presence of essential hypertension, mixed dyslipidaemia, and an impaired fasting glucose level. Physical examination showed a blood pressure of 143/104 mmHg, a heart rate of 89/min, a body weight of 80 kg, a height of 178 cm, and a BMI of 25.83 kg/m^2^. Laboratory tests indicated troponin levels of 27 ng/L, random glucose levels of 9.2 mmol/L, triglyceride levels of 2.72 mmol/L, and high-density lipoprotein (HDL) levels of 0.98 mmol/L. An ECG showed an elevated ST segment in I, aVL, and V1 to V5, and depressed in II, III, aVF, and V6. The timeline of patient care is shown in Figure 1.

### 2.2. Therapeutic Intervention

The patient was diagnosed with an anterior and lateral ST elevation myocardial infarction (STEMI) with a thrombolysis in myocardial infarction (TIMI) risk score of three. An urgent percutaneous coronary intervention (PCI) to the left anterior descending artery (LAD) was performed using a 3.5 × 20 mm Supraflex Cruz drug eluting stent (DES). The lesion was post-dilated with a 3.5 × 8 mm balloon at 14–16 atm, with a satisfactory angiographic result and no immediate complications. While waiting to be transferred back to the cardiac ward, the patient presented with an episode of ventricular fibrillation, which was immediately reverted with defibrillation in the cardiac catheterization laboratory. A post-resuscitation ECHO revealed left ventricular wall motion abnormalities, akinesia in the apex and anterior segment, hypokinesia in the inferior segment, and a preserved left ventricular ejection fraction of 50%. The ECG after cardioversion displayed no new ST segment elevation and the laboratory test showed no significant increased creatine kinase MB fraction (CK-MB).

### 2.3. Follow-Up and Outcomes

**Figure 2:**
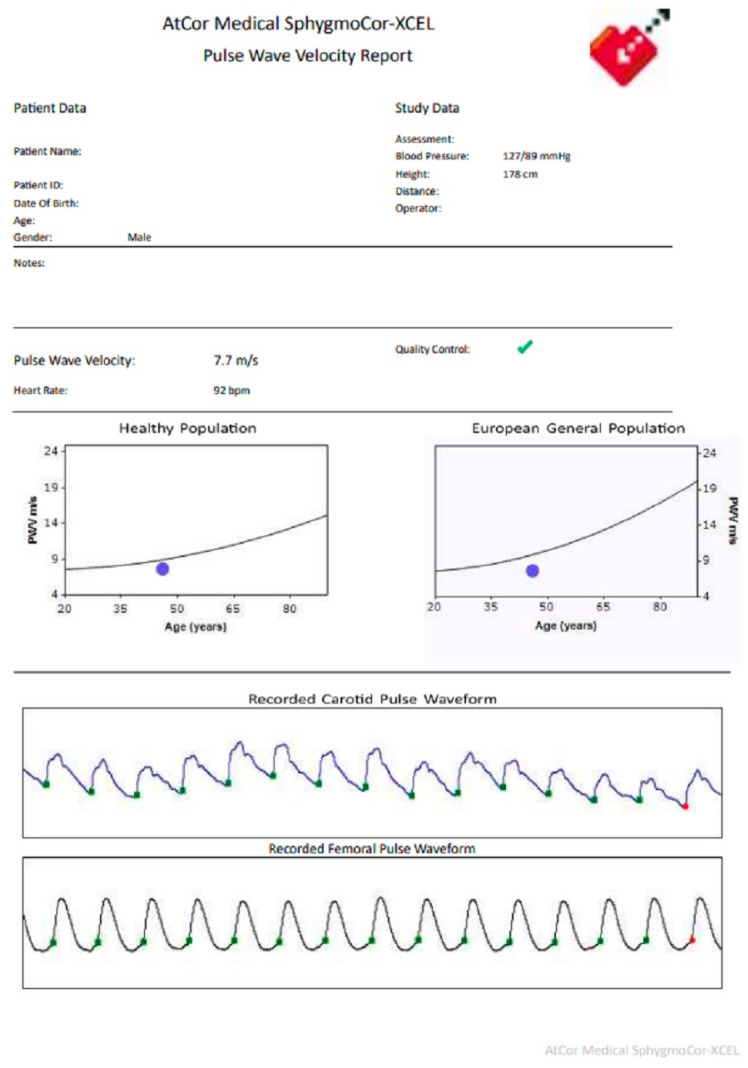
CfPWV report.

On day 8 post PCI, the patient remained asymptomatic and was discharged home with medications. The prescribed medications included aspirin 75 mg QD, ticagrelor 90 mg BD, proton pump inhibitor (PPI) 20 mg QD, metoprolol 23.75 mg QD, torsemide 5 mg QD, eplerenone 25 mg QD, and atorvastatin 20 mg QD. Six weeks after discharge, the patient was clinically admitted for the phase II cardiac rehabilitation program. The laboratory tests (Table 1) showed borderline traditional cardiovascular risk factors including a blood pressure of 113/74 mmHg, a heart rate of 87/min, fasting glucose levels of 5.9 mmol/L, glycated haemoglobin (HbA1c) levels of 5.8%, triglyceride levels of 1.89 mmol/L, and HDL levels of 1.06 mmol/L. CfPWV 7.7 m/s (Figure 2) was measured via SphygmoCor XCEL device (AtCor Medical Limited, Sydney, Australia) [9]. The patient was haemodynamically stable, chest pain free, and completed the phase II cardiac rehabilitation which consists of risk factors assessment and modification, exercise training, nutritional counselling, physical activities recommendation, and psychosocial management. In the physical exercise aspect, a 6 min walk test was performed and the patient complained of fatigue when travelling a distance of 540 m. The cardiac stress test indicated an exercise tolerance of less than three metabolic equivalents (METs). According to the Polish Cardiac Society guideline [10], the patient was categorized into model D of cardiac rehabilitation training. In the psychosocial aspect, the patient’s mood, emotion, mental tension, and perception of illness, work, and life goals were evaluated by a psychologist. Appropriate psychosocial interventions were provided to promote a positive health attitude. Also, the patient was reminded about the importance of dietary and lifestyle modifications to reduce the risk of future cardiovascular events. He continued the phase III cardiac rehabilitation program in the community.

## 3. Discussion

An increased PWV is associated with an elevated arterial stiffness, which alters myocardial perfusion and increases the risk of sudden death in patients of MetS. Kim et al. investigated 848,498 people with MetS who underwent health screenings, and showed that a high serum gamma-glutamyl transferase associated with an impaired aortic elasticity in MetS, which accounts for a 50% increased risk of sudden cardiac death [11]. Similarly, Li et al., contingent on the National Health and Nutrition Examination Survey, reported that obesity as a single MetS component increased the PWV and elevated 5.76-fold the risk of cardiovascular mortality [12]. Moreover, Masrouri also reported a MetS population with a 1.4- to 1.6-fold increased risk of cardiac sudden death [13]. All these studies proved that PWV is one of the key factors influencing cardiovascular risk in patients with subclinical MetS. Recently, Skrypnik et al. indicated that the implementation of the cardiac rehabilitation programme can lead to a decrease in serum leptin levels [14], which can influence arterial stiffness [15].

After the event of STEMI and sudden cardiac arrest (SCA), the presented case showed good control of traditional cardiovascular risk factors such as a blood pressure of 113/74 mmHg, a heart rate of 87/min, fasting glucose levels of 5.9 mmol/L, HbA1c levels of 5.8%, triglyceride levels of 1.89 mmol/L, and HDL levels of 1.06 mmol/L. However, we could not stratify the case as a low-risk patient, as atherosclerosis may be subclinical in asymptomatic MetS. Compared with the mean PWV of 5.4 m/s for the age range of 45 to 50 [16], the mean PWV of 7.7 m/s of the presented case may indicate low vessel compliance due to a long-term exposure to components of MetS. The study of Li et al. demonstrated a positive correlation between the estimated pulse wave velocity (ePWV) and all-cause mortality when the ePWV is greater than 6.7 m/s [12]. It is also important to note that a traditional 10 year Systematic Coronary Risk Evaluation (SCORE) scale may potentially underestimate cardiovascular risk in patients with diabetes, central obesity, low HDL, and elevated triglyceride levels [17,18]. For instance, the calculated SCORE risk scale for the presented case is 3.4% [19] which indicated a low risk of cardiovascular event. However, the presented case showed the non-classical risk factors like increased CfPWV, which increased the relative risk of cardiovascular disease. Thus, a screening for CfPWV could be useful in identifying a subclinical atherosclerosis and cardiovascular disease in asymptomatic populations with MetS.

To date, PWV is well recognised for evaluating arterial stiffness, predicting future cardiovascular event and all-cause mortality, and stratifying a high risk of subclinical atherosclerosis. CfPWV can be measured using different devices including tonometry, oscillometry, ultrasonography, and magnetic resonance imaging [20]. Among these techniques, pressure sensors like tonometers are considered as a low-cost method for measuring PWV [21,22]. For instance, a traditional imaging modality, such as a CT scanner or an MRI machine, costs a million dollars, while a CfPWV measuring device is far more affordable, with prices ranging from around USD 26,000 to USD 100,000. A CfPWV measuring device also has a relatively low operating cost compared with other imaging technologies. The device requires no expensive consumables such as contrast agents, and can be operated by a clinician or a trained nurse in a clinical consultation room. In addition, it only consists of a notebook computer, pressure cuffs, main measuring unit, and a pen-sized tonometer. It is easily accessible with no dedicated rooms or facilities required. Because of this, CfPWV measuring has been shown to be a cost-effective screening method in clinical practice.

Despite the fact that prevention is better than cure in terms of cost effectiveness and quality of life, CfPWV is not mandatorily integrated in clinical practice in Poland. The sub-optimised use of CfPWV screening in primary clinical practice is mainly due to the myth of a lack of methodological consensus and a lack of reproducibility consensus [23]. The European Society of Cardiology guideline regards CfPWV as the “gold-standard” to measure aortic stiffness and evaluate arterial system damage [24]. The normal reference values of CfPWV have been established in various countries to overcome the challenge of standardization and replication. The optimal cut-off point of CfPWV is essential to evaluate subclinical atherosclerosis and predict a risk of future cardiovascular events. The American Heart Association follows the European Society of Cardiology, European Society of Hypertension guidelines, and an expert consensus document, adopting 10 m/s as a cut-off value for CfPWV [25]. In Poland, a study from Podolec et al. suggested a CfPWV value greater than 11.7 m/s was significant, with a high risk of cardiovascular disease [26]. By applying these validated PWV cut-off values, clinicians are more convinced to categorize the level of arterial stiffness and to work out a quality care management system. The validation of a non-invasive arterial pulse wave velocity measurement device is another key issue to be considered when implementing CfPWV screening in clinical practice. Recently, some scientific societies [27] collaborated on providing a guideline for the methodology of validating a new device. The guideline indicated that well validated reference devices, like the Sphygmocor system or the Complior Analyse device, can be used to validate a new non-invasive device. However, it is also crucial for the PWV device developers to validate the technology which enhances the consistency and reliability of the CfPWV data.

The crucial clinical implication of measuring CfPWV is to estimate arterial stiffness and to inform a risk stratification for future cardiovascular events. The at-risk population includes those with coronary artery disease, chronic kidney disease, diabetes mellitus, hypertension, and dyslipidaemia [28]. The total economic costs of cardiovascular diseases in the European Union and Poland are EUR 282 billion and EUR 9.6 billion per year, respectively [29,30]. These financial burdens are attributable to metabolic risk factors that can be modified or controlled through targeted risk screening. For instance, measuring CfPWV is a simple procedure that can be conducted in clinical settings. In the present case, we did not perform a primary CfPWV screening as the patient was critically ill when first admitted to the emergency department. Yet, CfPWV screening was performed in the follow-up consultation. The CfPWV was obtained non-invasively via measuring the PWV between the right carotid and the right femoral artery through a well-validated SphygmoCor device in a quiet clinical room. The patient had abstained from food, tobacco, caffeine, and alcohol before the measurement. Anthropometric and blood pressure parameters, including body weight, body height, body mass index, and brachial blood pressure, were recorded in the device system. Then, the patient was arranged in a supine position. After entering the data, the carotid-femoral path length was calculated. Next, we applied the femoral cuff around the patient’s thigh and placed the tip of the tonometer on the patient’s right carotid pulse. The femoral cuff was inflated simultaneously with the high-quality carotid pulse wave form. After that, the CfPWV report was generated from the device system within a minute. In this connection, we considered CfPWV as safe, validated, and highly sensitive to a target participant who is at risk of subclinical atherosclerosis. Based on the increased CfPWV, the patient was reclassified to a higher cardiovascular risk group. Taking into account the drastic increase in the prevalence of MetS in the Polish population, a timely identification of a high cardiovascular risk group is beneficial to optimize a secondary preventive care strategy.

### Learning Points

1. The current case report demonstrated a positive relationship between MetS and SCA.
2. MetS increased irreversible arterial stiffness, which may be asymptomatic for patients with borderline conventional cardiovascular risk factors.
3. PWV provides additional risk information over the conventional cardiovascular risk stratification.
4. PWV screening can be relevant to identify subclinical atherosclerosis in asymptomatic population with MetS.

## 4. Conclusions

The current case report demonstrated that increased CfPWV in MetS correlated with STEMI and SCA. However, the case report should be interpreted with caution as SCA could also to be associated with post-revascularization reperfusion in this case report. Additional prospective research is needed to evaluate the role of CfPWV in clinical practice, body check-up, and medical follow-up for risk stratification.

## Figures and Tables

**Table 1:** Summary of laboratory data.

Parameters	In Emergency Department	In Follow-Up Clinic
Carotid-femoral pulse wave velocity (CfPWV) m/s	Not available	7.7
Body weight (kg)	80	78
Height (cm)	178	178
Body mass index (kg/m^2^)	25.83	25.2
Blood pressure (mmHg)	143/104	113/74
Heart rate (bpm)	89	87
Random glucose (mmol/L)	9.2	Not available
Fasting glucose (mmol/L)	Not available	5.9
Glycated haemoglobin (HbA1c)(%)	Not available	5.8
Triglyceride (mmol/L)	2.72	1.89
High-density lipoprotein (HDL)(mmol/L)	0.98	1.06
Low-density lipoprotein (LDL)(mmol/L)	1.94	2.38

## Data Availability

The datasets generated and/or analysed during the current study are available from the corresponding author on reasonable request.

## Abbreviations

BaPWV	Brachial-ankle pulse wave velocity
CfPWV	Carotid-femoral pulse wave velocity
DES	Drug eluting stent
ePWV	Estimated PWV
HDL	High-density lipoprotein
LAD	Left anterior descending artery
MetS	Metabolic syndrome
MET	Metabolic equivalents
PCI	Percutaneous coronary intervention
PWV	Pulse wave velocity
SCA	Sudden cardiac arrest
SCORE	Systematic Coronary Risk Evaluation
STEMI	ST Elevation Myocardial Infarction
TIMI	Thrombolysis In Myocardial Infarction
